# Transcriptome Analysis of *Ceriops tagal* in Saline Environments Using RNA-Sequencing

**DOI:** 10.1371/journal.pone.0167551

**Published:** 2016-12-09

**Authors:** Xiaorong Xiao, Yuhui Hong, Wei Xia, Shipeng Feng, Xi Zhou, Xiumei Fu, Jian Zang, Yong Xiao, Xiaolei Niu, Chunxia Li, Yinhua Chen

**Affiliations:** 1 Hainan Key Laboratory for Sustainable Utilization of Tropical Bioresource, Hainan University, Haikou, P.R China; 2 College of Agriculture, Hainan University, Haikou, P.R China; Huazhong University of Science and Technology, CHINA

## Abstract

Identification of genes involved in mangrove species’ adaptation to salt stress can provide valuable information for developing salt-tolerant crops and understanding the molecular evolution of salt tolerance in halophiles. *Ceriops tagal* is a salt-tolerant mangrove tree growing in mudflats and marshes in tropical and subtropical areas, without any prior genome information. In this study, we assessed the biochemical and transcriptional responses of *C*. *tagal* to high salt treatment (500 mmol/L NaCl) by hydroponic experiments and RNA-seq. In *C*. *tagal* root tissues under salt stress, proline accumulated strongly from 3 to 12 h of treatment; meanwhile, malondialdehyde content progressively increased from 0 to 9 h, then dropped to lower than control levels by 24 h. These implied that *C*. *tagal* plants could survive salt stress through biochemical modification. Using the Illumina sequencing platform, approximately 27.39 million RNA-seq reads were obtained from three salt-treated and control (untreated) root samples. These reads were assembled into 47,111 transcripts with an average length of 514 bp and an N50 of 632 bp. Approximately 78% of the transcripts were annotated, and a total of 437 genes were putative transcription factors. Digital gene expression analysis was conducted by comparing transcripts from the untreated control to the three salt treated samples, and 7,330 differentially expressed transcripts were identified. Using k-means clustering, these transcripts were divided into six clusters that differed in their expression patterns across four treatment time points. The genes identified as being up- or downregulated are involved in salt stress responses, signal transduction, and DNA repair. Our study shows the main adaptive pathway of *C*. *tagal* in saline environments, under short-term and long-term treatments of salt stress. This provides vital clues as to which genes may be candidates for breeding salt-tolerant crops and clarifying molecular mechanisms of salt tolerance in *C*. *tagal*. The expression levels of 20 candidate genes measured by RNA-Seq were validated via qRT-PCR. Eighteen genes showed consistent expression patterns in RNA-Seq and qRT-PCR results, suggesting that the RNA-seq dataset was dependable for gene expression pattern analysis.

## Introduction

As an environmental stressor, high salinity affects plants’ growth and productivity via several major physiological processes, including energy and fat metabolism, protein synthesis, and photosynthesis [1]. Plants adjust their growth and development to cope with salt stress, which can result in decreased yields or death. Approximately 20% of agricultural land worldwide has been affected by salinization, and over 50% of arable land is predicted to have hypersaline and alkaline soil by 2050 [2]. Salt damage is one of the main abiotic stresses limiting the growth and development of crops; thus, understanding how crops deal with salinization is vital to sustainable development of agriculture, under increasing environmental pressure.

Mangroves, a major contributor to the marine environments, are salt-tolerant trees that grow in mudflats and marshes in the tropics and subtropics. The growing conditions of mangroves include high salinity, UV radiation and temperature, low nutrient availability, and long-term hypoxia, all of which has provided selective pressure for effective active oxygen scavenging mechanisms [3]. Mangroves have a complex root system and salt filtration system to cope with wave action and salt water immersion [4]. The root systems of mangroves protect reefs from runoff of terrestrial sediments and other kinds of pollution, as well as providing a habitat for marine organisms [5]. Mangroves’ salt tolerance makes them ideal model organisms for studying plant adaptation to salt stress.

Mechanisms of salt tolerance in mangroves have been described in terms of morphology, anatomy, physiology, biochemistry [6]. Mangroves have developed a range of adaptations to cope with high salt stress, including ion compartmentation, specialized osmoregulation, and selective ion uptake and transport [4]. However, studies on the mechanisms of salt tolerance have been carried out on *Arabidopsis thaliana*, a small dicotyledonous species [7, 8], and *Mesembryanthemum crystallinum*, a facultative halophyte [9, 10]. Despite their ecological importance, mangroves’ molecular genetic adaptations to highly saline intertidal habitats have not been described in detail. Characterization of the molecular mechanisms of salt tolerance in mangroves, particularly identification of candidate genes involved in salt tolerance, will inform breeding and genetic engineering of salt-tolerant crops.

Because genomic and transcriptomic resources for mangroves are currently unavailable, in-depth genomic study of their salt-tolerance mechanisms has encountered a bottleneck. RNA sequencing (RNA-seq) is a high-throughput sequencing method that yields millions of short cDNA reads. RNA-seq is an effective tool for transcriptome analysis and it enables gene expression profiling even of single cells. RNA-seq has been widely used in transcriptome sequencing of model and non-model species, including *Arabidopsis thaliana* [11], *Pinus contorta* [12], *Eucalyptus grandis* [13], *Ipomoea batatas* [14], *Rhizophora mangle*, *Heritiera littoralis* [15], and *Sonneratia alba* [16].

Here, we used Illumina RNA-seq to sequence the transcriptome of *Ceriops tagal*, a mangrove tree species in the family *Rhizophoraceae* [17]. After *de novo* assembly of the resulting short-read sequences, predicted genes were annotated, despite of the absence of prior genome information for this species. Our results provide a molecular fundamental of discovering candidate salt-tolerant genes in *C*. *tagal*.

## Results and Discussion

### Biochemical modification of *C*. *tagal* under high salt stress

**Fig 1:**
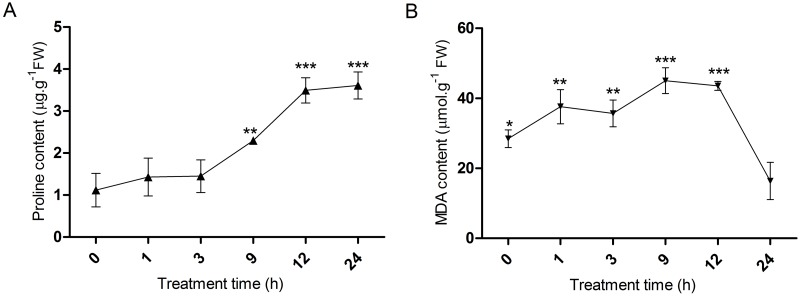
Variation in proline and malondialdehyde (MDA) contents through salt treatment time in *C*. *tagal* roots tissues. Root samples were collected from seedlings that were treated with 500 mmol/L NaCl. Three replicates were used for each treatment and data are presented as the mean ± standard deviation, * p < 0.05, as determined using Student’s *t*-test.

The major compatible solutes including proline are thought to function as osmoprotectants for protein [18]. Since proline plays a major role in protecting cells by stabilizing cell membranes and proteins [19], proline content was measured in root samples of *C*. *tagal* after salt treatment (Fig 1A). The proline content of roots increased significantly with increasing treatment time. The increase in proline content was slow in the early (0–3 h) and late stages (12–24 h) of salt treatment; but the value rose threefold in the middle stage (3–12 h). Thus, the active period of proline accumulation in *C*. *tagal* roots lasted from 3 to 12 h of salt treatment. Increasing proline levels in *C*. *tagal* roots under high salinity are similar to the response seen in other salt-tolerant plants, such as wilting perennial rye grass [20]. In plants, there are two pathways for synthesizing proline: ornithine and glutamate. The glutamate pathway is the main source of proline production under osmotic stress. Proline oxidase, which converts proline into glutamate, is the major regulator of accumulation of osmolytes [20]. Whether proline accumulation is a stress effect or one of the mechanisms of stress tolerance in *C*. *tagal* remains unclear.

Alterations in lipid metabolism and composition, along the presence of particular lipid species, have all been associated with environmental stress-mediated modifications of plant growth and development, including those related to high salinity [21]. Under adverse environmental conditions in plants, malondialdehyde (MDA) is one of the most prevalent byproducts of lipid peroxidation. To assess the extent of membrane damage during high salt stress, MDA content was measured in *C*. *tagal* roots (Fig 1B). The MDA content increased linearly in the first 12 h of salt treatment, and subsequently decreased over the next 12 h, to a level lower than the 0h treatment. This change in MDA content indicated that salt treatment initially caused oxidative stress in *C*. *tagal* roots; however, *C*. *tagal* recovered from the stress, differing in this response from salt-sensitive plants [22]. This suggests that *C*. *tagal* may have an intrinsic system to repair oxidative damage from salt stress. Changes in MDA levels can strongly induce gene expression changes. Previous research using structural mimics of MDA isomers demonstrated that their propensity for cross-linking or modifying reagents might facilitate the activation of gene expression. Changes in the concentration or localization of unbound MDA *in vivo* could strongly affect stress-related transcription [23].

### RNA-seq data and de novo assembly transcripts of *C*. *tagal*

To investigate transcriptional responses of *C*. *tagal* to salt stress, RNA-seq data was generated from seedling root tissues after 0, 1, 3, 9, 12, and 24 h of salt treatment. The Illumina run produced a total of 27,399,300 clean short reads, of 2,465,937,000 bp in total, with an average length of 90 bp (Table 1).

**Table 1:** Summary of RNA-seq data and *de novo* assembly transcripts for *C*. *tagal* root tissues.

	Number	Mean length (bp)	N50 (bp)	Total length (bp)
Read	27,399,300	90	90	2,465,937,000
Contig	99,262	283	380	28,069,303
Unigene	47,111	514	632	24,228,614

**Fig 2:**
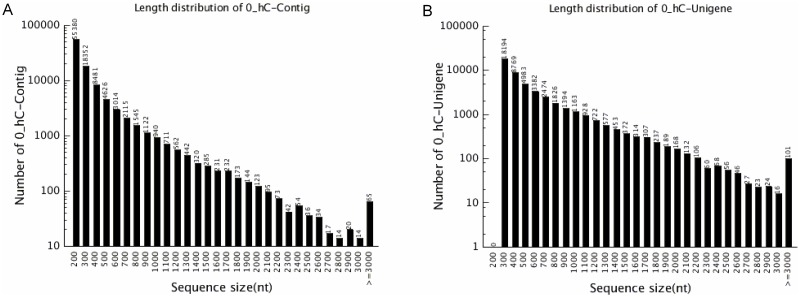
Statistical analysis of sequence length distribution, for the transcripts derived from Trinity *de novo* assembly of *C*. *tagal* short reads. (A) Size distribution of contigs. (B) Size distribution of unigenes.

Using Trinity *de novo* assembly, these short RNA-seq reads were assembled into 99,262 contigs and 47,111 unigenes (Fig 2A). Contig sequence length varied from 100 bp to over 3000 bp, with an average length of 283 bp. The number of contigs was reduced with increasing contig length. Contigs thought to be from the same transcript were linked and extended at either or both ends to obtain the longest unigenes possible, generating another 10,000 unigenes. In all 57,304 unigenes were generated, with an average length of 514 bp, with lengths ranging from 200 bp to over 3000 bp (Table 1 and Fig 2B). Additionally, 12.94% of the unigenes were longer than 1000 bp (the putative length for a complete gene). Thus, numerous transcripts (Bioproject ID PRJNA348664) for mangrove roots under normal and salt stress conditions were obtained, providing new information that can be used in studies of gene expression and function in mangroves.

### Functional annotation of *C*. *tagal* transcriptome

The assembled *C*. *tagal* unigenes were BLASTed against public databases to obtain functional annotations. Results from nr and Nt provided more unigene annotations than the other four databases, returning 35,191 and 32,262 unigene annotations, respectively. Together with the unigenes annotated using SwissProt (19,643), KEGG (17,872), COG (9,765), and GO (17,154), approximately 78% (36,741) of all assembled unigenes (47,111) were annotated. These functional annotations provided predicted biological functions and membership of and biosynthetic pathways, of the assembled unigenes.

**Fig 3:**
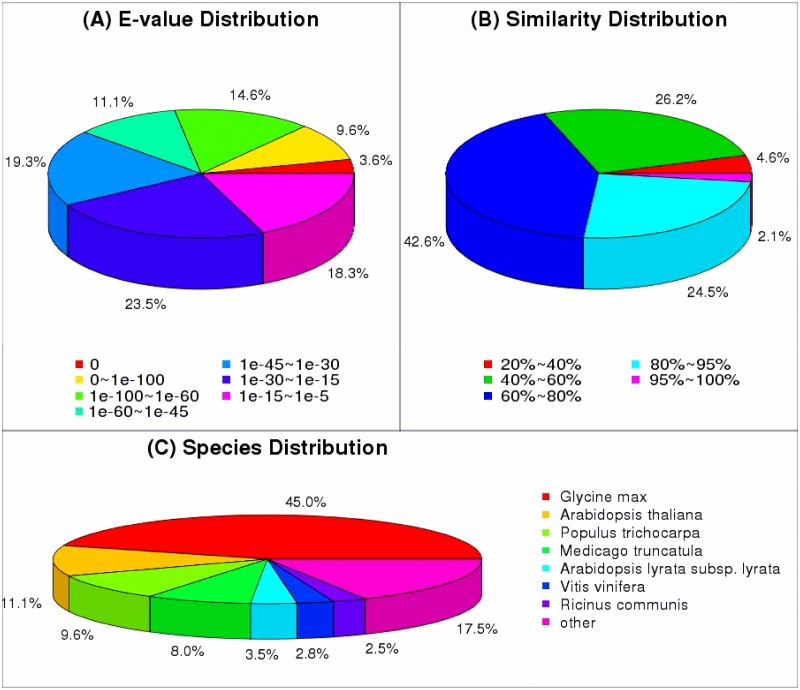
Comparison of *C*. *tagal* unigenes against the nr protein databases. (A) E-value distribution of the top BLAST hits for each unique sequence. (B) Similarity distribution of the top BLAST hits for each unique sequence. (C) Species distribution of the top BLAST hits for all homologous sequences.

BLAST results from the nr database indicated that 27.8% of alignments had an E value < 1e-60 (Fig 3A), while just over a quarter (26.6%) of unigenes had more than 80% similarity with known protein sequence (Fig 3B). Statistical analysis of alignments between *C*. *tagal* and seven model plant species showed that 45% of the top alignment results were from *Glycine max*, while 2.5–11.1% of alignments were to the other six species, when comparing with the nr database (Fig 3C). This suggests that the *C*. *tagal* genome is more closely associated with that of *G*. *max* than with the other model plant genomes. Based on this information, the genome of *G*. *max* could be regarded as a reference for molecular biological research in *C*. *tagal*.

Transcription factors (TFs) play important roles in plant development and stress tolerance [21]. In present study, 437 genes encoding putative TFs, in 55 TF families, were identified in *C*. *tagal* (S1 Table). Since these putative TFs were derived from RNA isolations from a single tissue type, the total number of putative TFs belonging to each TF family was lower than that seen genome-wide in *Thellungiella parvula* or *Arabidopsis thaliana*. Among these TF families, ethylene response factor (ERF, 35 putative TFs), MYB factor (28), bHLH (44), bZIP (28), and WRKY (27) contained the most TFs identified from the *C*. *tagal* root transcriptome.

MYB and WRKY families are thought to be involved in the abiotic stress response [24]. In maize, the expression of genes related to lignin synthesis is repressed by the overexpression of MYB31 and MYB42 [25], which manifests as an up to 45% reduction in lignin content, and substantially increased leaf, stem, and root growth. In the present study, 16 of 24 MYB genes were differentially expressed under salt treatment. Five of them (CL6067.Contig2, CL4864.Contig2, CL6599.Contig1, Unigene7236, and CL5514.Contig1) were upregulated in the early stages and downregulated after 9 h of salt treatment. The expression levels of eight MYB genes (CL712.Contig2, CL6592.Contig1, CL6686.Contig1, CL712.Contig1, Unigene6579, CL2898.Contig1, CL2353.Contig1, and CL2353.Contig2) showed a steady increase up to 24 h of salt treatment, while three members (Unigene267, CL2745.Contig1, and CL2933.Contig1) were downregulated. Expression of one member of the MYB TF family (Unigene6579) was approximately 20-fold higher than in controls, after 24 h of salt treatment; other cases of upregulation were approximately two times the expression level seen in the relevant control. This indicates that MYB genes might have a close relationship with stress tolerance in plants by affecting lignin synthesis.

The WRKY transcription factors are thought to repress the gibberellin (GA) signaling pathway, activate the abscisic acid (ABA) signaling pathway [26], and regulate several other signaling pathways in plants [27]. The GA and the ABA are important regulators of the salt stress response. In this study, twenty-one putative WRKY transcription factors were differentially expressed, between the treated samples and the control of *C*. *tagal* root tissues. Nine genes (CL5729.Contig1, CL757.Contig5, CL6730.Contig1, Unigene5897, CL1691.Contig1, CL5811.Contig2, CL6730.Contig2, CL5729.Contig2, and CL4863.Contig2) were upregulated over the course of 24h treatment; meanwhile, ten (CL179.Contig1, Unigene2977, CL4141.Contig2, Unigene5746, Unigene6567, CL4457.Contig2, CL2949.Contig1, Unigene6165, CL1264.Contig1, and Unigene2517) were upregulated over the course of 9h treatment but downregulated after 12h treatment. Two genes showed relatively low expression levels and were further downregulated throughout the treatment period. Six genes (CL757.Contig5, CL6730.Contig1, Unigene6567, CL1264.Contig1, Unigene2517, and CL179.Contig1) were more than eight-fold upregulated compared to the control (Table 2). These genes were involved in the GA and ABA signal transduction pathways. These findings imply that GA and ABA might affect stress tolerance in plants by participating in signaling pathways.

**Table 2:** Differential expression of MYB and WRKY transcription factors (TF) in the root of *C*. *tagal* under salt stress.

TF family	Upregulated genes (17)	Downregulated genes (5)	Upregulated earlier, downregulated later (15)
MYB	CL712.Contig2, CL6592.Contig1, CL6686.Contig1, CL712.Contig1, Unigene6579, CL2898.Contig1, CL2353.Contig1, CL2353.Contig2	Unigene267, CL2745.Contig1, CL2933.Contig1	CL6067.Contig2, CL4864.Contig2, CL6599.Contig1, Unigene7236, CL5514.Contig1
WRKY	CL757.Contig5, CL6730.Contig1, Unigene5897, CL1691.Contig1, CL5811.Contig2, CL6730.Contig2, CL5729.Contig2, CL4863.Contig2, CL5729.Contig1	CL4827.Contig3, Unigene4792	CL4141.Contig2, Unigene5746, Unigene6567, CL4457.Contig2, CL2949.Contig1, Unigene6165, CL1264.Contig1, Unigene2517, CL179.Contig1, Unigene2977

### Identification and characterization of differentially expressed genes under salt stress

In this study, digital gene expression (DGE) library sequencing was used to explore the expression of salinity-responsive unigenes in *C*. *tagal* root. Four DGE libraries were constructed and sequenced, from *C*. *tagal* roots treated with salt for 0, 1, 9, or 24 h; 7.36, 8.78, 7.29, and 7.81 million reads were generated, respectively. Up to 50% of the assembled unigenes were detected by DGE reads (Table 3).

**Table 3:** Summary of DGE datasets from the root of *C*. *tagal* under salt stress.

Summary	Number of reads (percentage)			
	0 h*	1 h	9 h	24 h
Clean reads (total)	7,364,032 (100.00%)	8,783,461 (100.00%)	7,288,190 (100.00%)	7,814,807 (100.00%)
Mapped reads	3,488,211 (47.36%)	3,856,370 (43.90%)	3,571,681 (43.91%)	3,627,844 (46.42%)
Reads with perfect matches	2,922,774 (39.69%)	3,222,871 (36.69%)	3037804 (41.68%)	3,043,187 (38.94%)
Reads with unique matches	2,851,117 (38.72%)	2,771,639 (31.55%)	2,555,456 (35.06%)	2,617,561 (33.49%)

*0, 1, 9, and 24 h represent root samples from *C*. *tagal* seedlings at 0, 1, 9, and 24 h of salt treatment (500 mmol/L NaCl), respectively.

**Fig 4:**
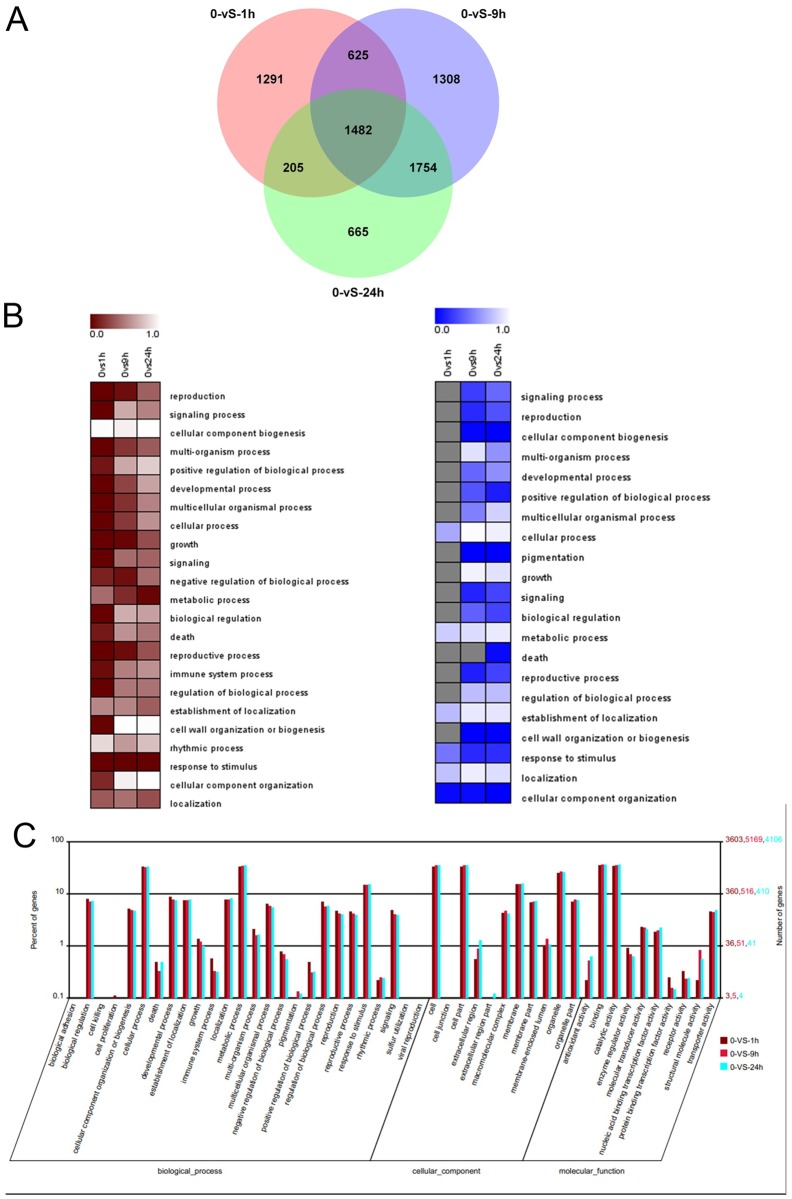
Characterization of differentially expressed genes from the root of *C*. *tagal* at 1, 9, and 24 h of salt treatment. (A) Venn diagram of digital gene expression (DGE) libraries for 0-VS-1 h, 0-VS-9 h, and 0-VS-24 h. (B) The biological process of gene ontology (GO) enrichment analysis for DGEs. Left: Upregulated genes. Right: Downregulated genes. Columns and rows indicate treatments and biological process GO terms, respectively. (C) Histogram of gene ontology classifications for DGEs. The left-hand y-axis indicates the percentage of annotated DGEs from 1, 9, and 24 h in each category; the right-hand y-axis indicates the number of annotated DGEs from 1, 9, and 24 h.

In comparison to the control (0h exposure to salt stress), the 1, 9, and 24h samples had 3,603, 5,169, and 4,106 differentially expressed unigenes, respectively. In total, 7,330 unigenes were detected as being differentially expressed in one or more of the 1,9, and 24h samples, while 1482 unigenes were commonly expressed among all three samples (Fig 4A). Further comparison of unigene expression patterns between the three sampling stages showed that more differentially-expressed genes were uniquely shared between 9 and 24 h (1,754) than between 1 and 9 h (625) or 1 and 24 h (205). This suggests that there is a difference between short-term and long-term responses of gene expression to the salt stress treatment.

For functional categorization of these 7,330 unigenes, gene ontology (GO) terms were assigned to each unigene. GO analysis demonstrated that upregulated genes were involved in 23 biological processes and downregulated genes were involved in 21 biological processes (Fig 4B). Multiple biological processes that are active under salt stress being up- or downregulated suggests that *C*. *tagal* has a complex response to high salinity conditions. The distribution of unigenes at each of the three different treatment times was similar among groups (Fig 4C).

### Patterns of dynamic gene expression under salt stress

**Fig 5:**
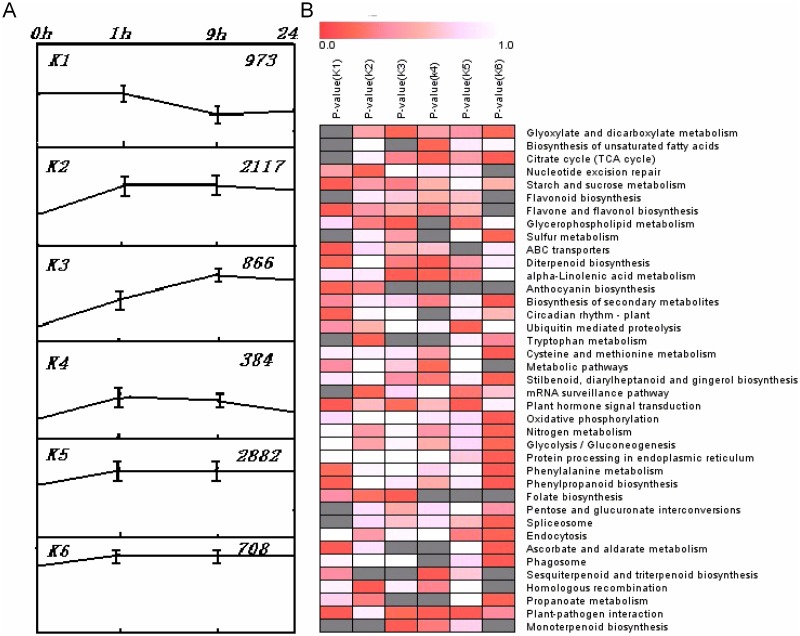
Complete k-means cluster maps and genes in each k-means cluster with KEGG annotation. (A) K-means clustering showing the expression profile of digital gene expression. (B) Pathway enrichment among the six clusters. Columns and rows indicate each cluster genes and GO terms, respectively. Color scales indicate p values of enrichment tests and gray represents missing values.

To further classify unigenes that were responsive to salt stress, k-means clustering was adapted to group the all differentially expressed unigenes (7,330) into six clusters (K1, K2, K3, K4, K5, and K6) based on the pattern of expression changes across the four sampling stages (0, 1, 9, and 24 h). Among these six clusters, K5 (2882) and K2 (2117) included the most unigenes, while K4 (384) had the fewest. Unigenes belonging to clusters K2, K3, K5, and K6 showed increasing expression levels through time, while unigenes in K1 showed decreasing expression through time and expression of those in K4 initially rose and subsequently dropped (Fig 5A).

To evaluate which biological pathways the unigenes in clusters K1–K6 belong to, further annotation was done using KEGG (Fig 5). In K1, expression levels decreased to 9 h and then remained constant up to 24 h. Genes in this cluster were annotated as being involved in plant-pathogen interaction, plant hormone signal transduction, ascorbate, and aldarate metabolism. In K1, UNIGENE3688, CL3293.CONTIG2, and UNIGENE2870 are putative auxin response factors, a pathway important in regulating plant growth under stress [28]. UNIGENE269, CL227.CONTIG2, CL3810.CONTIG1, CL747.CONTIG2, and CL747.CONTIG1 were all annotated as the DELLA protein, which is a critical repressor in the GA signaling pathway, and is involved in hormonal or environmental signaling that regulates plant development [29]. Annotations of other genes participating in stress responses were also found in this cluster, including respiratory burst oxidase (CL4559.CONTIG), L-ascorbate oxide (CL5522.CONTIG1, UNIGENE4947, CL2848.CONTIG1, UNIGENE1041, CL5020.CONTIG1, UNIGENE4580, CL4920.CONTIG2, UNIGENE4183, and UNIGENE3265), and flavonoid 3′-monooxygenase (UNIGENE2668, UNIGENE3033, and CL4404.CONTIG1).

In cluster K2, expression initially rose sharply up to 1 h, but was then constant up to 9 and 24 h. The genes annotated in this cluster are mainly involved in homologous recombination, tryptophan metabolism, and nucleotide excision repair processes. UNIGENE4096 and UNIGENE2106 were annotated as recombination protein RecA, and DNA excision repair protein ERCC-2, respectively. UNIGENE4927 and CL5148.CONTIG1 were annotated as RAD54 (DNA repair and recombination protein RAD54 and RAD54-like protein).

The expression of genes in cluster K3 rose from 1 to 9 h, and remained constant up to 24 h. Genes in this cluster belong to monoterpenoid biosynthesis, folate biosynthesis, and alpha-linolenic acid metabolism pathways. CL6362.CONTIG2 was annotated as allene oxide cyclase, which is important in defense reactions and stress responses. CL2381.CONTIG1 and CL2529.CONTIG2 were putatively annotated as jasmonate O-methyltransferase, which participates in the synthesis of methyl jasmonate and also regulates development and stress responses [30].

In cluster K4, expression was higher at 1 h than at 9 h (both of which had higher expression levels than controls) and expression decreased to below control levels at 24 h. Genes in this cluster are involved in sesquiterpenoid, triterpenoid and diterpenoid biosynthesis, and plant-pathogen interactions. CL2844.CONTIG2 and CL2348.CONTIG1 were annotated as the BAK1 gene, which plays an important role in stress defense [31]; CL2399.CONTIG1, CL2187.CONTIG2, CL2932.CONTIG2, and CL2932.CONTIG3 were annotated as being homologues of MYC2, which is a transcription factor in the jasmonic acid signaling pathway [32].

The expression pattern for cluster K5 was similar to that of K4, except that the gene expression level stayed the same between 1 and 9 h. Genes in K5 were annotated as being involved in plant-pathogen interaction, plant hormone signal transduction; and ubiquitin mediated proteolysis, which alone had a rise in expression at 1 h and then remained constant between 9 and 24 h. CL3369.CONTIG1, CL1963.CONTIG2, CL1963.CONTIG5 and CL2392.CONTIG1 were annotated as HSP90; CL339.CONTIG2 was annotated as homologous to WRKY29, which is involved in stress defense [33].

Genes in cluster K6 participate in biological processes such as biosynthesis of secondary metabolites, phenylpropanoid biosynthesis, and phenylalanine metabolism. Expression levels in this cluster reached their highest point at 1 h and then remained constant through the rest of the treatment period. CL2970.CONTIG3 was identified as a NADH gene, which could remove active oxygen [34]. UNIGENE5590 and UNIGENE5743 were annotated as L-ascorbate oxidase.

### Expression pattern of putative genes involved in proline metabolism pathway

**Fig 6:**
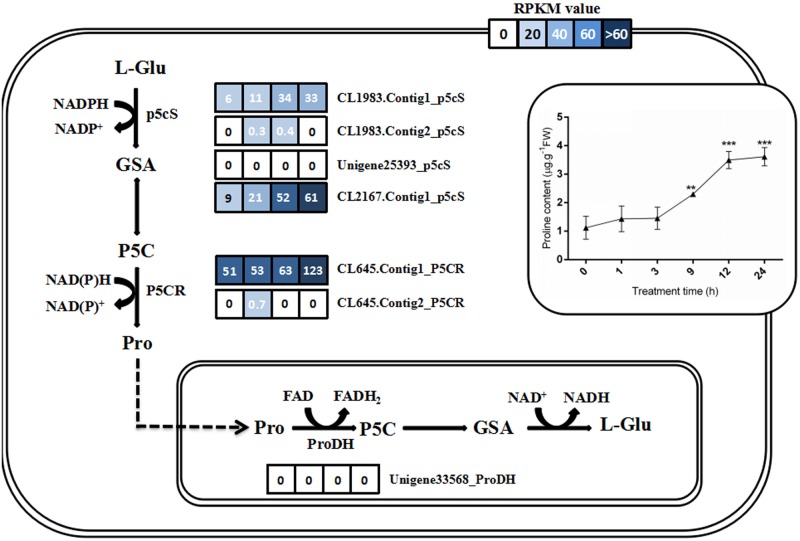
The expression patterns of seven unigenes putatively involving in the biosynthesis and metabolism of proline under salt treatment. Gene expression levels are indicated with the colored bars. The number within the color bar indicates the RPKM value at 0, 1, 9, and 24 hours of salt treatment.

In plant, proline is a crucial factor which balances osmotic stress. In the study, we investigate the gene expression change involving in the proline biosynthesis and metabolism (Fig 6). Four genes, CL1983.Contig1, CL1983.Contig2, Unigene25393, and CL2167.Contig1, showed high similarity with the known sequence of *P5CS* (Pyrroline-5-carboxylate synthase) that is the key enzyme in proline biosynthesis and responsible for converting glutamate into P5C. The previous study had reported that the enzyme gene was significantly induced under environmental stresses. Two *P5CS* genes (CL1983.Contig1 and CL2167.Contig1) were inducible expressed at 9 hour and 24 hour after salt treatment, in accordance with the change of proline content after salt treatment (Fig 6). However, the other two *P5CS* genes showed very low expression in four time points after salt treatment (Fig 6). Moreover, two genes, including CL645.Contig1 and CL645.Contig2, showed high similarity with the known sequence of *P5CR* (Pyrroline-5-carboxylate reductase), which is the last step of proline biosynthesis. After 24-hour salt treatment, the RPKM value of CL645.Contig1 was increased to 123, indicating that *P5CR* could be induced by high salt (Fig 6). These expression results indicated that proline would participate in osmotic stress adjustment for *C*. *tagal* surviving under high salt environment.

### Biosynthesis and metabolism of malonaldehyde (MDA) under salt stress in *C*. *tagal*

**Fig 7:**
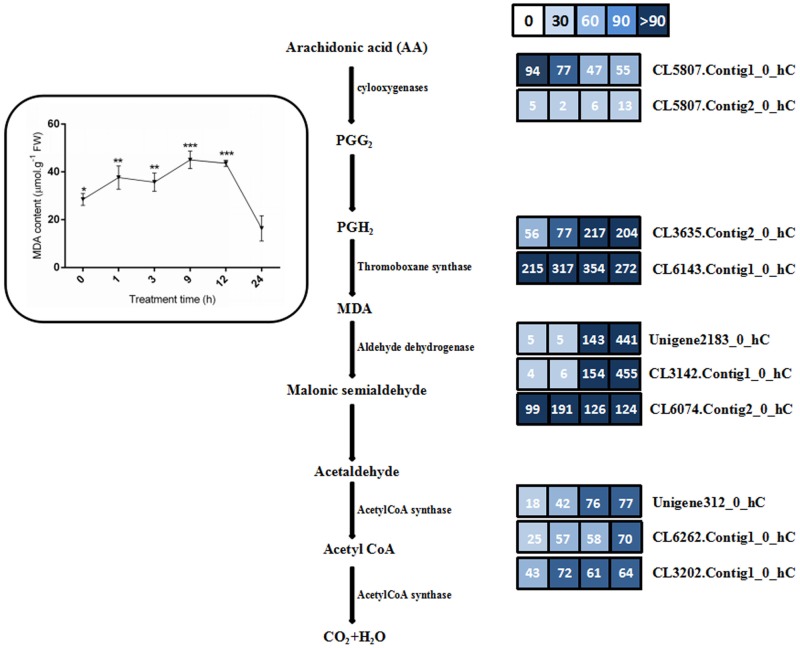
The expression patterns of ten unigenes putatively involving in the biosynthesis and metabolism of MDA under salt treatment. Gene expression levels are indicated with the colored bars. The number within the color bar indicates the RPKM value at 0 hour, 1 hours, 9 hours, and 24 hours after the salt treatment.

MDA is one of the most prevalent byproducts of lipid peroxidation under adverse environmental conditions in plants. In this study, we investigated the expression of genes involved in the biosynthesis and metabolism of MDA (malonaldhyde). Based on the transcriptome data, a total of 4 expressed sequences were identified as cylooxygenases (CL5807.Contig1 and CL5807.Contig2) and Thromoboxane synthase (CL3635.Contig2 and CL6143.Contig1), which are involved in MDA synthesis. As showed in Fig 7, the four genes, except CL5807.Contig1, were inducible expressed. after salt treatment, indicating that lipid peroxidation was activated under salt stress. Meanwhile, six unigenes were identified to have high similarity with the known gene sequence of aldehyde dehydrogenase (unigene2183, CL3142.Contig1, and CL6074.Contig 2) and acetylCoA synthase (unigene312, CL6262.Contig1, and CL3202.Contig1), which are involved in degradation and metabolism of MDA (malonaldehyde).These six genes were strong induced at 9 hour and 24 hour after salt treatment (Fig 7). These data indicates that MDA can be degraded and metabolized to decrease MDA damage in *C*. *tagal* under salt stress.

### Verification of RNA-seq data

**Fig 8:**
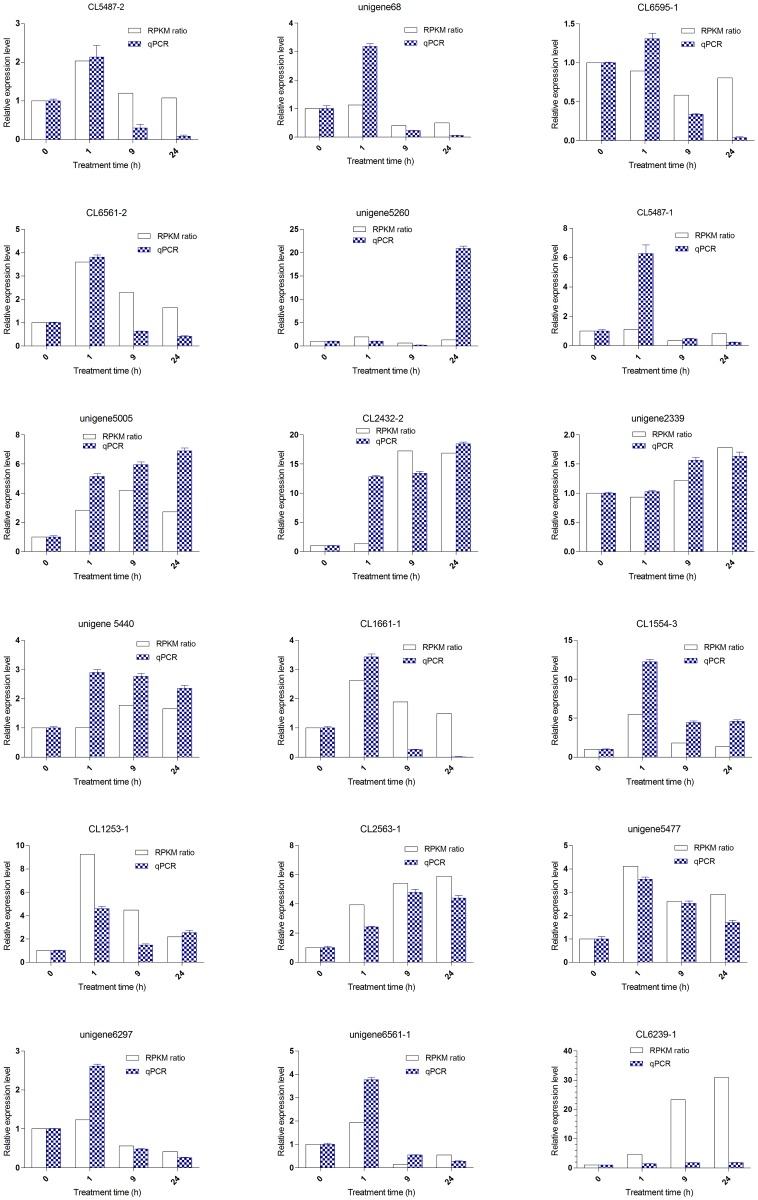
qRT-PCR validation of differentially expressed genes in the root of *C*. *tagal* after salt treatment with 500 mmol/L NaCl. Twenty candidate genes were chosen at random. Small-subunit (18S) ribosomal RNA was used as a control, with its expression levels set to one. The expression levels of all other samples were normalized to the levels of the control. A: Transcription factors. GRAS (CL5487-2, unigene68, CL6595-1, and CL6561-2) have the same expression pattern, that increased at 1 h of treatment and down to the level of control; ARF (unigene5260) increased at the time point of 24 h; scarecrowe-like protein-like (CL5487-1) increased at 1 h of treatment and down at the other point; B: Four of salt-response related genes (unigene5005, CL2432-2, unigene2339, and unigene5440) increased all the time, and three of them (CL1661-1, CL1554-3, and CL1253-1) increased at 1 h; C: Predicted proteins without annotation (CL2563-1, unigene5477, unigene6297, CL6561-1 and CL6239-1).

Real-time quantitative reverse transcription PCR (qRT-PCR) was used to validate the expression levels measured by RNA-seq for 20 candidate genes, chosen at random. Two of the 20 candidate genes showed no amplification; all of the remaining 18 genes tested showed consistent expression patterns (up- or downregulation at the same time point) in the RNA-seq and qRT-PCR results (Fig 8). The absolute value of the expression level of each of three genes varied between the RNA-seq and qRT-PCR data. DGE analysis indicated a change of expression level of CL6239.contig1 from 5× to 30× higher than the control, between 1 and 24 h, while no obvious differential expression was detected for the same gene by qRT-PCR. Expression of CL5487.contig1 and unigene5260 in qRT-PCR results increased by 6× from 0 to 1 h, and by 20× to 24 h. These results suggest that the RNA-seq dataset obtained for *C*. *tagal* roots was dependable for analyzing gene expression patterns.

## Conclusions

In this study, *C*. *tagal*, a plant adapted to high salinity, was analyzed for biochemical and transcriptional changes, to understand its response to salt stress. In *C*. *tagal* root tissues, proline accumulation early means mangrove plants could survive salt stress through biochemical modification including osmotic substance accumulation. Meanwhile, malondialdehyde content increased progressively from 0 to 9 h, then dropped to below control levels by 24 h of salt treatment. This implied that *C*. *tagal* plants could adapt to high salt habitat. Using RNA-seq and DGE analysis, a comprehensive description of changes in gene expression was developed. Among the differentially expressed genes annotated, 7330 putative stress response genes were identified, 166 genes were deduced to be transcription factors, 160 genes were thought to be involved in signal transduction, and 58 were ATPase-related genes. Complex gene responses to salt stress were revealed. In clusters K1–K6, DNA repair-related genes were shown to be rapidly upregulated to cope with the DNA damage caused by salt stress, while some other genes appeared to have a role in stress defense across all stages of salt treatment. All these results provide fundamental information for selection of candidate genes in breeding salt-tolerant crops, as well as clarifying molecular mechanisms of salt stress adaptation in *C*. *tagal*.

## Materials and Methods

### Plant materials and high salt treatment

Hypocotyls of mature *Ceriops tagal*, with no signs of pathology, were closely matched in terms of developmental maturity, length, and individual weight. The hypocotyls were immersed in Hoagland nutrient solution for cultivation. When the second pair of leaves began budding, seedlings were transferred into a 500 mmol/L NaCl solution. Control seedlings were placed in 0 mmol/L NaCl. Seedlings were left in their respective solution for one of 0, 1, 3, 9, 12, or 24 h. Each treatment was performed in triplicate, 5 seedlings in each triplicate. Physiological and molecular indicators in the root of *C*. *tagal* were measured after treatment.

### Estimation of proline and malondialdehyde contents

Free proline content was estimated following the method of Bates et al. (1973). Fresh root tissue (0.5 g) was extracted in 4 mL of 3% sulphosalicylic acid. A total of 2 mL of the supernatant was reacted with 2 mL of acid ninhydrin and 2 mL of glacial acetic acid in a test tube for 1 h at 100°C; the reaction was then terminated in an ice bath. The reaction mixture was extracted with 4 mL of toluene and vortexed for 15–20 s. The chromophore-containing toluene was aspirated from the aqueous phase and warmed to room temperature before the absorbance was measured at 520 nm using a SpectraMax^®^ Plus spectrophotometer (Molecular Devices, Sunnyvale, CA, USA). Proline concentration was calculated from a standard curve using 0–50 μg L-proline (Sigma).

Lipid peroxidation was measured as the amount of malondialdehyde (MDA) produced by the thiobarbituric acid reaction [35]. Fresh roots (0.2 g) of control and NaCl-treated plants were homogenized in 2 mL of 20% (w/v) trichloroacetic acid. The homogenate was centrifuged at 3500 g for 20 min and the supernatant was collected. Next, 1.5 mL of 20% trichloroacetic acid containing 0.5% (w/v) thiobarbituric acid, and 100 μL 4% butylated hydroxytoluene in ethanol were added to a 0.5 mL aliquot of the supernatant. This mixture was heated at 95°C for 30 min and was then quickly cooled in ice before centrifugation at 10,000 g for 15 min. The absorbance of the supernatant was measured at 532 nm by spectrophotometry. Non-specific absorption was subtracted. The concentration of MDA was calculated using an extinction coefficient of 155 mmol L^−1^cm^−1^.

### RNA extraction, cDNA library preparation, and RNA-seq

Total RNA was extracted from roots using the modified cetyltrimethylammonium bromide-sour phenol extraction method [36]. RNA was digested with RNase-Free DNase (Qiagen, Venlo, Limburg, Netherlands) and checked by capillary gel electrophoresis for integrity. Oligo(dT) beads (Beyotime, Shanghai, China) were used to isolate poly(A) mRNA. Long mRNA was cut into short fragments in the fragmentation buffer. First-strand cDNA was synthesized using random-hexamer primers, with the short fragments as templates. Buffer containing dNTPs, RNaseH, and DNA polymerase I was used to synthesize second-strand cDNA according the Second Strand cDNA Synthesis Kit instructions (Beyotime, Shanghai, China). Short fragments were purified using the QiaQuick PCR extraction kit (Qiagen), using Ethidium bromide buffer for end repair and addition of poly(A). Sequencing adapters were then added to the short fragments. After agarose gel electrophoresis, suitable fragments were selected for the PCR amplification as templates. The cDNA library was sequenced using an Illumina HiSeq^™^ 2000 (Illumina Inc., San Diego, California, USA).

### *De novo* transcriptome assembly

Transcriptome assembly was performed using Trinity [37] following the protocol proposed by Grabherr et al. (2011), which adopts a k-mer graph approach to assemble Illumina RNA-seq reads. Trinity was used to combine short reads into longer sequence fragments, initially based on a specific overlap length. These assembled sequences, or contigs, were then joined to further short reads using pair-end joining and gap-filling. A minimum of three read pairs was used as the standard to define the order and distance between two contigs, in order to avoid misassembling chimeric reads. Contigs from the same transcript could be detected with paired-end reads. Assembled contigs were combined to obtain unigenes, defined as longer sequences that could not be further extended on either end.

### Annotation and categorization of unigenes

The assembled *C*. *tagal* unigenes were BLASTed against the NCBI nr, Swiss-Prot, KEGG, and COG databases, with an e-value cut-off of 1e-5. BLAST results were used to annotate putative unigene functions, assigning function based on the protein match with the highest sequence similarity, in each case. The databases were ordered NCBI nr, then Swiss-prot, when the BLAST results conflicted between different databases. Unigenes that had successfully aligned to nr (higher priority) were not BLASTed against Swiss-Prot (lower priority). Alignments were considered complete when all databases had been checked. Coding region sequences were then determined for predicted proteins with the highest ranks using BLAST. When no matches were found for unigenes in any of the above databases, ESTScan software [38] was used for gene model prediction, in order to determine the nucleotide (5′–3′) and amino acid sequences of predicted coding regions.

GO annotation of unigenes was performed using the Blast2GO program [39], results of which were used for KEGG and COG analysis [40]. GO functional classification of the unigenes was performed using WEGO (http://wego.genomics.org.cn) to display the distribution of gene functions [41]. BLASTall was used for COG and KEGG pathway annotations, BLASTing against the COG and KEGG databases.

### Data analysis

Genes that were upregulated or downregulated at each time point were subjected to GO enrichment analysis, using the agriGO web interface (http://bioinfo.cau.edu.cn/agriGO/index.php). GO terms were selected to display as mapped to their locations in the GO tree (GO level 3).

A total of 2,023 TFs from Arabidopsis (belonging to 55 TF families) were downloaded from the plant transcription factor database (http://planttfdb.cbi.edu.cn/index.php?sp=At). These downloaded TFs were compared with 25,097 protein coding genes of *Thellungiella parvula* and assembled unigenes of *C*. *tagal*, using BLAST to generate alignments.

Reads per kb per million reads (RPKM) was used for calculating gene expression levels based on RPKM = 10^9^C / NL, where C is the number of reads solely aligned to one expressed sequence, N is the total number of reads simultaneously aligned to all expressed sequences, and L is the basic number in the coding sequence of the corresponding expressed sequence. The K-Means Clustering module in the Genesis software (http://genome.tugraz.at/genesisclient/genesisclient_description.shtml) was used to generate clusters. Expression trends for each of four time points (0, 1, 9, and 24 h) were based on log_2_RPKM values (RPKM values between 0 and 1 were assigned a value of 0 when clustering). K-means clusters were based on all differentially-expressed genes.

### Real-time quantitative RT-PCR verification of RNA-seq data

To verify the differential expression detected by Illumina RNA-seq, qRT-PCR was performed on the same RNA extractions that had been used previously. A set of 20 genes was chosen at random, each of which would be checked at different time points and compared to for detecting at different time points with their observed RPKM. qRT-PCR was performed by using an ABI 7500 Real-Time instrument thermocycler (Applied Biosystems, Foster City, CA, USA) and the Light Cycler fast-start DNA Master SYBR Green I kit (Roche, Basel, Switzerland). Reactions contained 0.5 μL of each primer (10 μmol/L), 100 ng of cDNA, and 10 μL SYBR Green Master Mix in a final volume of 20 μL. Reactions were performed in triplicate. Amplification was performed as follows: 95°C for 5 min, followed by 40 cycles of 95°C for 15 s, 60°C for 20 s, and 72°C for 30 s. Melting curve analysis was performed by raising the temperature from 55°C to 95°C (0.5°C per 10 s); gel electrophoresis confirmed the presence of single amplicons in the final product. Relative fold differences were calculated using the 2^−ΔΔC*t*^ method [42]. Small-subunit (18S) ribosomal rRNA was used as a control gene. The level of gene expression obtained from RNA-seq data were shown as the ratio of RPKM relative to 0 h treatment, which was set at a value of 1, to compare the expression levels measured by RNA-seq and qRT-PCR.

## Supporting Information

S1 Table Putative transcription factors identified in *Ceriops tagal* and comparison with *Thellungiella parvula* and *Arabidopsis thaliana*. (DOC)
