# Supplementary material for: Transcriptome Analysis of Ceriops tagal in Saline Environments Using RNA-Sequencing
Source: PLoS One. 2016 Dec 9;11(12):e0167551. doi: 10.1371/journal.pone.0167551 (PMC5147905; doi:10.1371/journal.pone.0167551)
Supplement: S1 Table — (DOC) [file pone.0167551.s001.doc]

**S1 Table. Putative transcription factors identified in *Ceriops tagal* and comparison with** ***Thellungiella parvula* and *A*rabidopsis *thaliana.***

| **Gene family** | **Definition** | **Number of genes** | | |
| --- | --- | --- | --- | --- |
|  |  | ***C. tagal*** | ***T. parvula*** | ***A. thaliana*** |
| AP2 | APETALA2 protein involved in cell development | 3 | 17 | 25 |
| ARF | Auxin response factor | 3 | 16 | 32 |
| ARR-B | Response regulators, contain a Myb-like DNA binding domain called ARRM (type B) | 3 | 11 | 16 |
| B3 | B3 DNA binding domain, includes LAV, REM, and RAV family | 5 | 41 | 71 |
| BBR/BPC | BASIC PENTACYSTEINE is a regulator of the homeotic genes and BBR is a characterized nuclear localization signal | 2 | 6 | 11 |
| BES1 | BRI1-EMS-SUPPRESSOR 1 gene, accumulates in the nucleus in response to BRs | 2 | 6 | 8 |
| C2H2 | Zinc finger domains are relatively small protein motifs which contain multiple finger-like protrusions | 23 | 83 | 104 |
| C3H | A protein containing a Cys3His zinc finger domain | 22 | 41 | 56 |
| CAMTA | Calmodulin binding transcriptional factors | 3 | 6 | 7 |
| CO-like | CONSTANS like genes contain two conserved domains—zinc finger region and CCT domain | 4 | 15 | 19 |
| CPP | Cystein-rich polycomb-like protein involved in cell division | 2 | 6 | 10 |
| DBB | Double B-box zinc fingergenes | 2 | 8 | 12 |
| Dof | DNA binding with one finger genes family | 8 | 31 | 43 |
| E2F/DP | E2F transcription factors are a family of proteins that share a related DNA-binding domain, and most E2F proteins associate with a DP protein and form heterodimeric complexes that bind to DNA in a sequence-specific manner | 3 | 8 | 12 |
| EIL | Ethylene-insensitive like protein | 2 | 6 | 6 |
| ERF | Ethylene response factors | 35 | 111 | 132 |
| FAR1 | Far-red impaired responsive family protein | 14 | 17 | 20 |
| G2-like | Golden 2-like genes | 8 | 40 | 55 |
| GATA | Light responsive transcriptional factors | 9 | 28 | 31 |
| GRAS | GAI, RGA, SCR gene family involved in plant developmental regulation | 10 | 29 | 36 |
| GRF | Growth regulation factors | 2 | 8 | 9 |
| GeBP | Noncanonical leucine-zipper transcription factors that regulate cytokinin response | 4 | 9 | 23 |
| HB-PHD | Homeobox proteins involved in cell differentiation and control of cell Growth, as well as patterning of diverse organisms | 2 | 2 | 2 |
| HB-other |  | 5 | 6 | 8 |
| HD-ZIP | Proteins that contain homeodomain and a leucine zipper motif immediately downstream of the homeodomain | 12 | 45 | 56 |
| HRT-like | A novel DNA-binding protein that contains three unusual zinc fingers with a CX8-9CX10CX2H consensus sequence | 1 | 2 | 2 |
| HSF | Heat stress factors | 9 | 29 | 25 |
| LBD | A new class of DNA-binding transcription factors that recognize the cis-element GCGGCG | 6 | 33 | 43 |
| LSD | Protein that contains three zinc finger domains, defined by CxxCxRxxLMYxxGASxVxCxxC | 2 | 3 | 6 |
| M-type | MADS-box TFs; 32-33, MYB: a family of proteins that include the conserved MYB DNA-binding domain | 2 | 31 | 73 |
| MIKC |  | 4 | 37 | 66 |
| MYB | A family of proteins that include the conserved MYB DNA-binding domain | 28 | 126 | 159 |
| MYB_related |  | 20 | 50 | 85 |
| NAC | NAM, ATAF, and CUC transcription factors that contain a highly conserved N-terminal DNA-binding domain and a variable C-terminal domain | 19 | 82 | 135 |
| NF-X1 | A type of zinc finger protein required for growth under salt stress | 2 | 2 | 2 |
| NF-YA | Nuclear binding factors Y transcription factor complex is composed of three unique subunits: NF-YA, NF-YB, and NF-YC | 3 | 10 | 15 |
| NF-YB |  | 4 | 12 | 19 |
| NF-YC |  | 5 | 9 | 15 |
| Nin-like | NIN proteins have regional similarity to transcription factors and function in nitrogen-controlled development | 2 | 14 | 14 |
| RAV | RAV family proteins contain a B3 domain, in addition to the single AP2/ERF domain | 1 | 5 | 6 |
| S1Fa-like | An unusual small peptide of only 70 amino acids, which contains a nuclear localization signal and a putative DNA binding helix | 1 | 1 | 4 |
| SAP | STERILE APETALA, a new class of transcription regulators essential in flower development | 1 | 1 | 1 |
| SBP | SQUAMOSA promoter binding proteins related to flower development | 3 | 13 | 18 |
| SRS | SHI RELATED SEQUENCE proteins with a single zinc finger motif | 2 | 10 | 12 |
| STAT | Signal transduction and activator of transcription | 1 | 2 | 2 |
| TALE | Transcription activator-like effector | 5 | 20 | 23 |
| TCP | Proteins sharing the so-called TCP domain, a 59-amino acid basic helix-loop-helix motif | 6 | 23 | 28 |
| Trihelix | Plant transcriptional activator that contains trihelix DNA-binding domains | 15 | 27 | 33 |
| VOZ | Vascular plant one zinc finger protein | 1 | 2 | 2 |
| WOX | The homeobox transcription factor superfamily characterized by the presence of a conserved DNA-binding homeodomain | 2 | 13 | 17 |
| WRKY | Proteins that contain the WRKY amino acid signature near the N-terminus | 27 | 67 | 89 |
| Whirly | Transcription factors ubiquitous in plant cell bonded to both ssDNA and RNA | 2 | 3 | 4 |
| ZF-HD | Zinc finger homeodomain protein involved in the establishment of the characteristic expression pattern of the C4 PEPCase gene | 3 | 15 | 17 |
| bHLH | Basic helix-loop-helix DNA-binding superfamily protein | 44 | 139 | 194 |
| bZIP | bZIP domain consists of two structural features located on a contiguous alpha-helix | 28 | 67 | 101 |
| Total |  | 437 | 1444 | 2014 |

Note: The transcriptional factors of *Arabidopsis* and *T. parvula* were downloaded from the database of plant transcriptional factors (<http://planttfdb.cbi.edu.cn/index.php?sp=At>)
